# The PROMIS-16 reproduces the PROMIS-29 physical and mental health summary scores accurately in a probability-based internet panel

**DOI:** 10.1007/s11136-024-03662-8

**Published:** 2024-04-23

**Authors:** Ron D. Hays, Patricia M. Herman, Anthony Rodriguez, Mary Slaughter, Chengbo Zeng, Maria Orlando Edelen

**Affiliations:** 1 Division of General Internal Medicine and Health Services Research, UCLA Department of Medicine, 1100 Glendon Avenue, Suite 850, Los Angeles, CA 90024 USA; 2 RAND Corporation, Behavioral and Policy Sciences, 1776 Main Street, Santa Monica, CA USA; 3 RAND Corporation, Behavioral and Policy Sciences, 20 Park Plaza #910, Boston, MA USA; 4 Patient Reported Outcomes, Value and Experience (PROVE) Center, Department of Surgery, Brigham and Women’s Hospital, Boston, MA USA

**Keywords:** PROMIS-29, PROMIS-16, Physical health summary, Mental health summary

## Abstract

**Purpose:**

The Patient-Reported Outcomes Measurement Information System® (PROMIS)-16 assesses the same multi-item domains but does not include the pain intensity item in the PROMIS-29. We evaluate how well physical and mental health summary scores estimated from the PROMIS-16 reproduce those estimated using the PROMIS-29.

**Methods:**

An evaluation of data collected from 4130 respondents from the KnowledgePanel. Analyses include confirmatory factor analysis to assess physical and mental health latent variables based on PROMIS-16 scores, reliability estimates for the PROMIS measures, mean differences and correlations of scores estimated by the PROMIS-16 with those estimated by the PROMIS-29, and associations between differences in corresponding PROMIS-16 and PROMIS-29 scores by sociodemographic characteristics.

**Results:**

A two-factor (physical and mental health) model adequately fits the PROMIS-16 scores. Reliability estimates for the PROMIS-16 measures were slightly lower than for the PROMIS-29 measures. There were minimal differences between PROMIS physical and mental health summary scores estimated using the PROMIS-16 or the PROMIS-29. PROMIS-16 and PROMIS-29 score differences by sociodemographic characteristics were small. Using the PROMIS pain intensity item when scoring the PROMIS-16 produced similar estimates of physical and mental health summary scores.

**Conclusion:**

The PROMIS-16 provides similar estimates of the PROMIS-29 physical and mental health summary scores. The high reliability of these scores indicates they are accurate enough for use with individual patients.

## The PROMIS-16 reproduces the PROMIS-29 physical and mental health summary scores accurately in a probability-based internet sample

The Patient-Reported Outcomes Measurement Information System® (PROMIS®)-29 v2.1 is a state-of-the-science health-related quality of life (HRQOL) profile measure [1]. The PROMIS-29 v2.1 assesses pain intensity using a single 0–10 numeric rating item and 7 health domains (physical function, fatigue, pain interference, depression, anxiety, ability to participate in social roles and activities, and sleep disturbance) using 4 polytomous (5 response categories) items per domain. The PROMIS-29 physical and mental health summary scores are created by multiplying PROMIS-29 domain scores by their factor scoring coefficients (available upon request from help@healthmeasures.net), adding these products together, and transforming the sum to the PROMIS T-score metric (mean of 50 and SD of 10 in the U.S. general population) [2]. The physical health summary score is a weighted combination of (in order of largest to smallest weight) physical function, pain (intensity and interference), ability to participate in social roles and activities, fatigue, emotional distress (anxiety and depression), and sleep disturbance; the mental health summary score is a weighted combination of fatigue, emotional distress, ability to participate in social roles and activities, pain, sleep disturbance, and physical function.

While the PROMIS-29 provides reliable and valid information about core aspects of HRQOL [3, 4], some researchers and clinicians elect to use the PROMIS global health measure because its ten items reduce the response burden of the PROMIS-29 by 66% [5]. The PROMIS global health measure is useful for general surveillance and risk adjustment but does not provide clinically actionable HRQOL domain scores.

In response to the need for a very brief PROMIS profile measure, Edelen et al. [6] developed the PROMIS-16 which will be available in the future at https://www.healthmeasures.net/. The PROMIS-16 assesses the same seven multi-item domains as the PROMIS-29 plus PROMIS cognitive function. Unlike the PROMIS-29, the PROMIS-16 does not include the pain intensity item. The eight domains of the PROMIS-16 are assessed using two items each. Eleven of the items are also included in the PROMIS-29; the other five items include one for the ability to participate in social roles and activities, two items for sleep disturbance, and two items for cognitive function. Edelen et al. [6] noted the need to evaluate how well the PROMIS-16 reproduces the PROMIS-29 physical and mental health summary scores. In this study, we estimate the physical and mental health summary scores using the existing PROMIS-29 factor scoring coefficients and the PROMIS-16 domain scores with and without the pain intensity item and compare those to the PROMIS-29 summary scores.

## Methods

### Sample

The English-language survey was made available for 10 days between September and October of 2022 to 7224 members of Ipsos’s KnowledgePanel, an internet probability-based panel designed to represent the general U.S. population. A total of 4,149 surveys were returned. We planned to screen out anyone who completed the survey with a response time of less than one second per item. None of the respondents met that criterion. We excluded the 0.5% who endorsed one or both of two fake conditions (“Syndomitis” or “Chekalism”) included within a list of chronic health conditions to identify careless or insincere respondents [7]. This resulted in an analytic sample of 4130 adults (57% response rate). Only 0.4% of the 4130 adults had missing data for more than half the items.

### Measures

The PROMIS-29 v2.1 was administered to estimate the PROMIS-29 physical health and mental health summary scores. The items in the PROMIS-16 not included in the PROMIS-29 v2.1 were also administered. We estimated the physical and mental health scores using PROMIS-16 estimated scores with and without the pain intensity item. A previous study showed that the PROMIS-29 mental health summary scores correlated more strongly with the 4-item PROMIS global physical health score than with the 4-item PROMIS global mental health score [8]. We administered the PROMIS global health items to assess whether summary scores estimated from the PROMIS-16 yielded a similar pattern of correlations.

All these PROMIS measures are scored on a T-score metric (mean = 50 and SD = 10 in the U.S. general population except for the sleep disturbance scale where the norm is a combination of the general population and a clinical sample), with higher scores representing better health.

### Human subjects protection

Study participants provided electronic consent upon starting the survey. All procedures were reviewed and approved by the research team’s Institutional Review Board (RAND Human Subjects Research Committee FWA00003425; IRB00000051).

### Analysis plan

We perform a maximum likelihood confirmatory factor analysis to assess the fit of the PROMIS-16 with and without pain intensity to the two correlated latent variables (physical and mental health) underlying the six measures included in the scoring of the PROMIS-29: physical function, pain composite, ability to participate in social roles and activities, fatigue, emotional distress, and sleep disturbance. We imposed the same structure and parameters on the PROMIS-16 (with and without pain intensity) multi-item measures as those used in the creation of the PROMIS-29 summary scores. Chi-square is reported but it is expected to be significant given the large sample size. Hence, we evaluate the practical fit of the model using the comparative fit index (CFI) and the root mean square error of approximation (RMSEA). CFI values of 0.95 or larger indicate good fit, and RMSEA values less than 0.09 represent acceptable fit [9, 10]. Then, we compute the standard PROMIS-29 physical and mental health scores and estimate summary scores using the PROMIS-16 with and without pain intensity. For all score evaluations, we focus primarily on results for the PROMIS-16 alone but also show values for the score versions that include the pain intensity item.

We report internal consistency reliability [11] for the PROMIS-16 and PROMIS-29 multi-item measures and Mosier’s composite reliability [12] for the physical and mental health summary scores. We evaluate these reliability indices using conventional criteria: acceptable: 0.70–0.79; good: 0.80–0.89; excellent: ≥ 0.90 [13]. We compare the reliabilities of PROMIS-16 measures with those estimated from the corresponding PROMIS-29 measures using the Spearman-Brown prophecy formula [14]. We also provide product-moment correlations among the measures and mean differences between them. In addition, we estimate intraclass correlations (random effects model) between corresponding measures [15]. We provide Bland–Altman plots [16] with the average of the PROMIS-16 and PROMIS-29 physical and mental health summary scores. The 95% upper and lower limits of agreement (bias) are estimated using: mean ± SD (mean difference) * 1.96. Scatter bias is present when the amount of disagreement varies by the average of the two estimates. Finally, we compute product-moment correlations of differences in PROMIS-29 and PROMIS-16 estimated physical and mental health summary scores with age, gender, education, and income. We also estimated correlations with the PROMIS global physical and mental health scores.

Analyses were performed using SAS 9.4 (TS Level 1M7) [17] and Mplus 8.7 [18] software.

## Results

### Sample characteristics

The ages of those in the sample (n = 4130) ranged from 18–94 with a median age of 54 and more people 60 and older than in younger age groups (Table 1). Forty-nine percent were female, and < 1% were transgender or did not identify as female, male, or transgender. Seventy percent were non-Hispanic White, 12% were Hispanic, 10% non-Hispanic Black, 5% non-Hispanic of another race, and 3% were two or more races. Thirty-four percent reported a high school degree or less, 26% had some college, and 40% had a bachelor’s degree or higher. Annual income was $100,000 or more for 42% of the sample.

**Table 1:** Sample Characteristics (n = 4,130)

Variable	Frequency	Percentage
**Age**		
18–29	560	14
30–44	953	23
45–59	911	22
60 or older	1706	41
**Gender**		
Female	2035	49
Male	2049	49
Transgender	11	< 1
Do not identify with the above	17	< 1
**Race/ethnicity**		
Hispanic	497	12
White	2887	70
Black	414	10
Another race	195	5
Two or more races	137	3
**Education**		
No high school	279	7
High school or GED	1097	27
Some college	1087	26
Bachelor’s degree	909	22
Master’s degree or higher	758	18
**Income (annual)**		
< $10,000	121	3
$10,000–49,999	1006	24
$50,000–99,999	1258	30
$100,000 or more	1745	42

### PROMIS-16 confirmatory factor analyses

The confirmatory factor analysis model estimated for the PROMIS-16 (without pain intensity) was rejectable statistically (χ^2^ (12 df = 391.532, p < 0.0001) but fit the data reasonably well: CFI = 0.97 and RMSEA = 0.09. The estimated correlation between physical and mental health was 0.46. Similar results were found for the confirmatory factor analysis model estimated for the PROMIS-16 with pain intensity included in the pain composite indicator. It was rejectable statistically (χ^2^ (12 df = 355.194, p < 0.0001) but fit the data reasonably well: CFI = 0.97 and RMSEA = 0.08. The estimated correlation between physical and mental health was 0.45.

### PROMIS-16 and PROMIS-29 reliabilities

Table 2 provides internal consistency reliability estimates for the multi-item scales and reliability estimates for the summary scores that are weighted combinations of the PROMIS-16 and PROMIS-29 measures. PROMIS-16 reliabilities ranged from 0.73 (sleep disturbance) to 0.95 (mental health summary score) while PROMIS-29 reliabilities ranged from 0.87 (sleep disturbance) to 0.98 (mental health summary score).

**Table 2:**
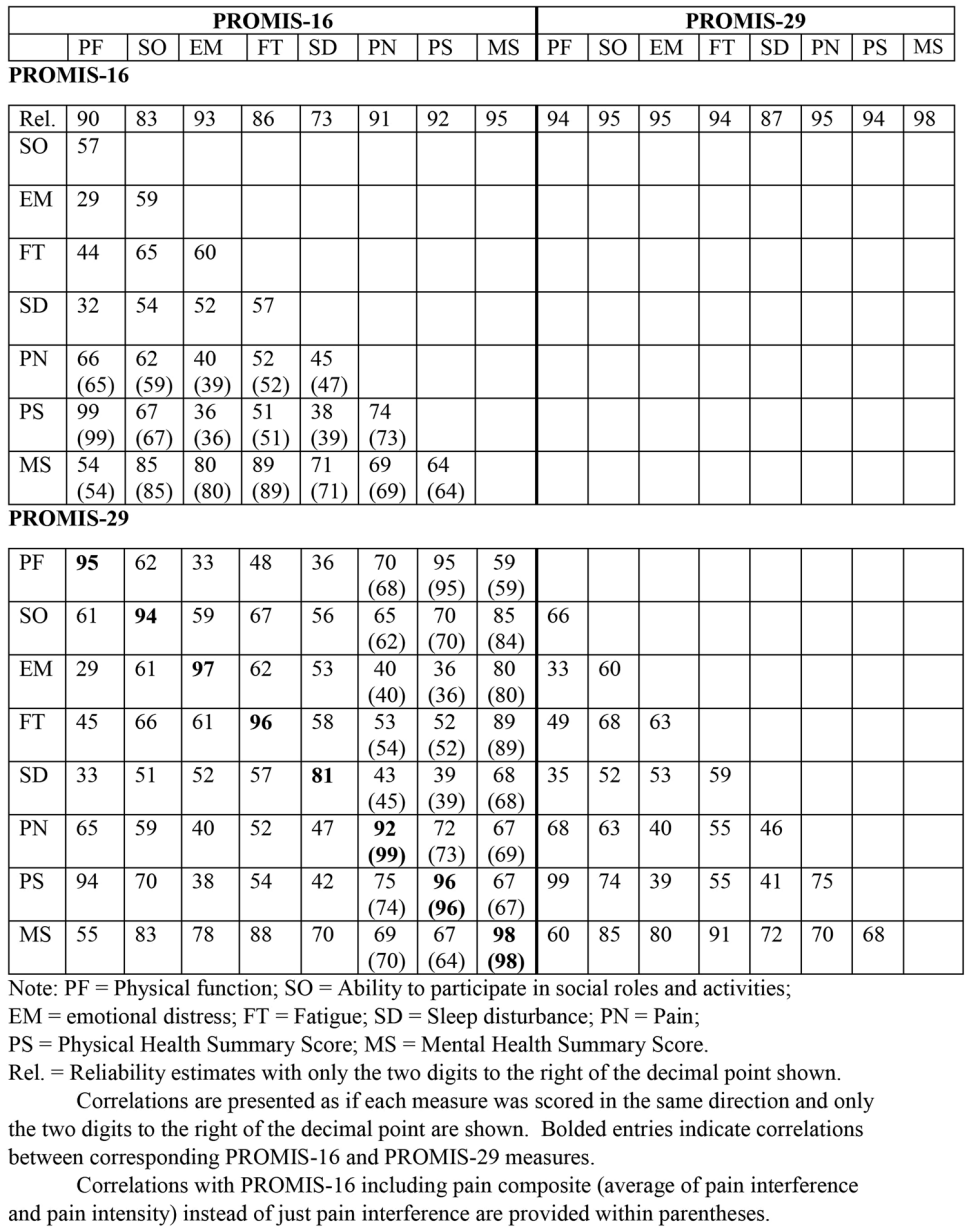
Product-moment Correlations Among the PROMIS-16 and PROMIS-29 Measures

The average difference between the reliabilities of the PROMIS-16 measure and those estimated from the Spearman-Brown prophecy formula was zero for the four measures used in creating the PROMIS physical and mental health summary scores that were a subset of the corresponding PROMIS-29 measures. However, the reliabilities for the two PROMIS-16 measures with some unique items were 0.04 (sleep disturbance) and 0.07 (ability to participate in social roles and activities) lower than those estimated from the PROMIS-29.

### Product-moment correlations among the PROMIS-16 and PROMIS-29 measures

Correlations among the PROMIS-16 and PROMIS-29 measures are shown in Table 2. Correlations within parentheses are provided to show the similarity of associations when the PROMIS pain measure used is the average of pain interference and the pain intensity item. The bolded entries in the correlation matrix, representing corresponding PROMIS-16 and PROMIS-29 measures, ranged from 0.81 (sleep disturbance) to 0.98 (mental health summary score). The correlations are all essentially perfect when adjusted for the unreliability of measurement.

### Product-moment correlations of PROMIS summary scores with the PROMIS global physical and mental health scores

The PROMIS-16 and PROMIS-29 physical health summary scores correlated more strongly with the PROMIS global physical health score (r’s = 0.73 and 0.75, respectively) than with the PROMIS global mental health score (r’s = 0.35 and 0.37, respectively). However, as expected based on previous research [8], the PROMIS-16 and PROMIS-29 mental health summary scores correlated more strongly with the PROMIS global physical health score (r’s = 0.72 and 0.75, respectively) than with the PROMIS global mental health score (r’s = 0.65 and 0.66, respectively).

### Mean differences between PROMIS-29 and PROMIS-16 measures

Mean scores and mean differences for the PROMIS-29 and PROMIS-16 are given in Table 3. The effect size differences ranged from 0.02 to 0.10 in absolute value. Intraclass correlations between corresponding PROMIS-29 and PROMIS-16 measures ranged from 0.81 (sleep disturbance) to 0.995 (pain).

**Table 3:** Mean Differences Between PROMIS-29 and PROMIS-16 Measures

Measure	PROMIS-29 Mean	PROMIS-16 Mean	Pooled SD	PROMIS-29–PROMIS-16 [Effect Size]	Intraclass Correlation
Physical function	50.54	51.71	8.14	−0.18 [−0.02]	0.94
Ability to participate in social roles and activities	55.79	54.90	8.62	0.89 [0.10]	0.93
Emotional distress	48.56	49.22	8.45	−0.66 [−0.08]	0.96
Fatigue	47.79	47.19	9.64	0.61 [0.06]	0.96
Sleep disturbance	48.54	48.78	8.09	−0.24 [−0.03]	0.81
Pain	50.10 (50.10)	50.00 (50.44)	8.58 (8.72)	0.09 [0.01] −0.34 [−0.04]	0.995 (0.995)
Physical health summary score	51.18 (51.19)	51.24 (51.21)	8.31 (8.31)	−0.06 [−0.01] −0.19 [−0.02]	0.96 (0.96)
Mental health summary score	52.85 (52.15)	52.64 (52.57)	8.25 (8.26)	0.21 [0.03] 0.27 [0.03]	0.98 (0.98)

The entries within parentheses in the bottom 4 rows indicate estimates using pain composite instead of pain interference

**Fig. 1:**
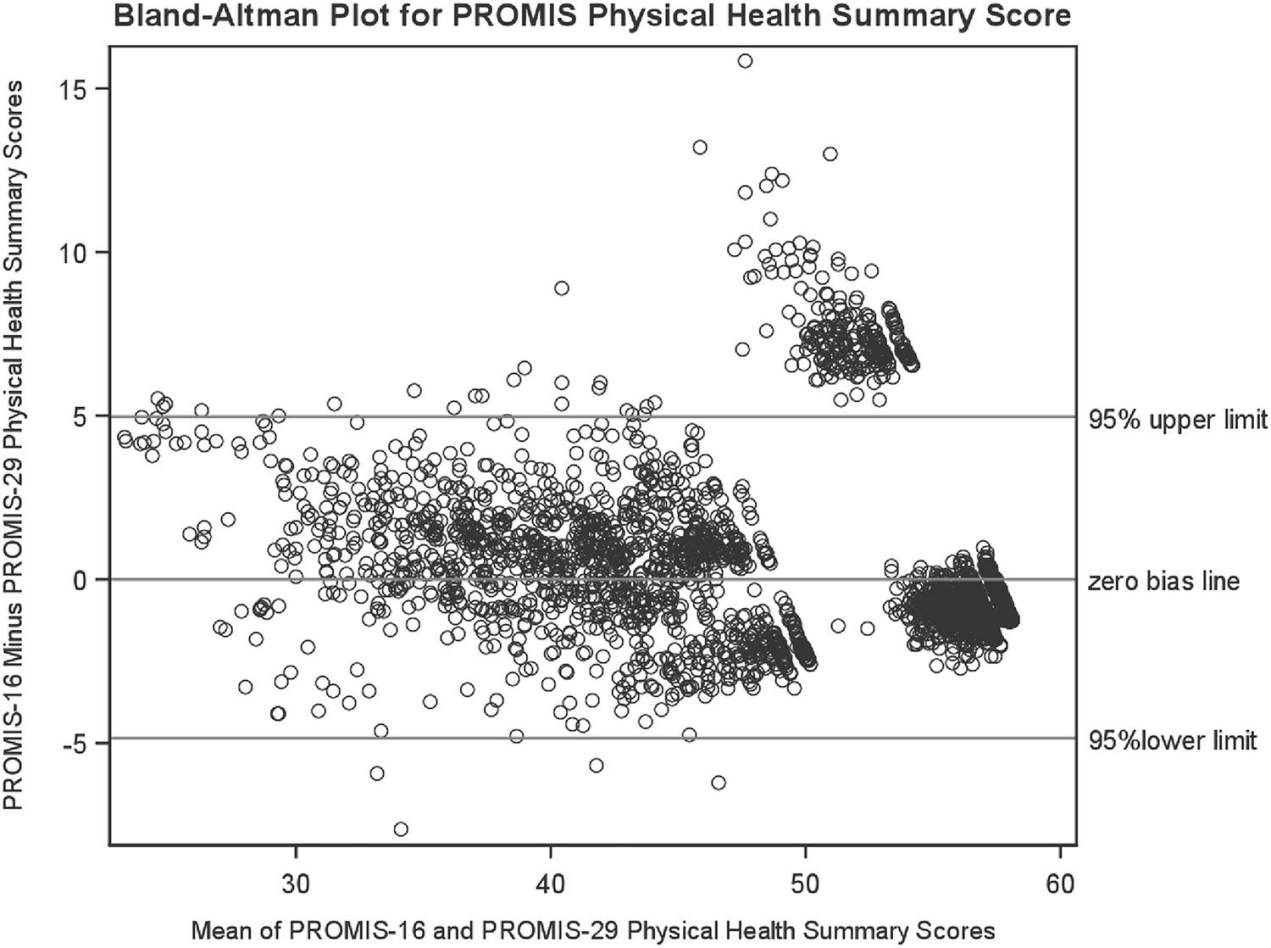
Bland–Altman Plot for PROMIS-16 Versus PROMIS-29 Estimated Physical Health Summary Score

**Fig. 2:**
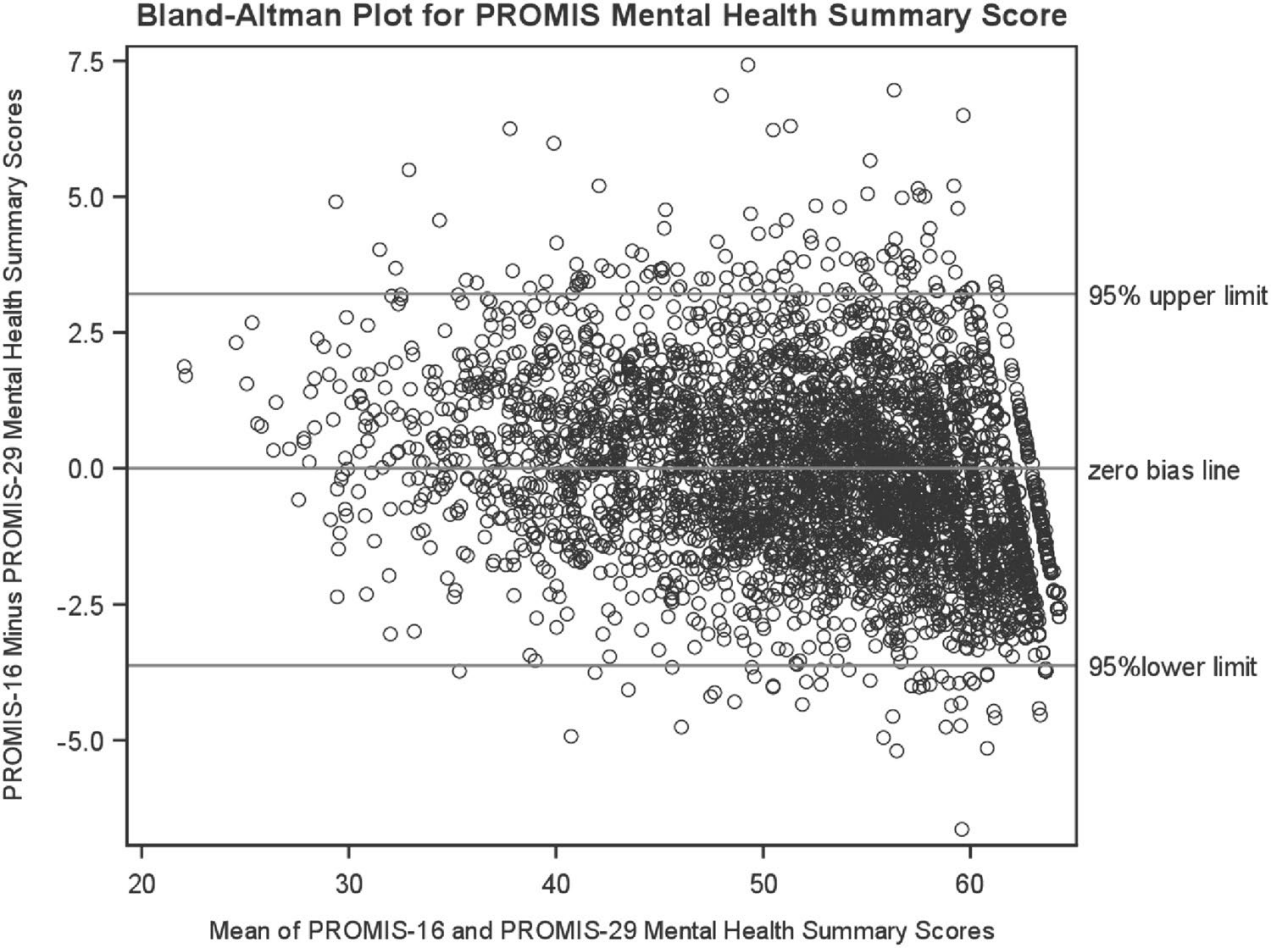
Bland–Altman Plot for PROMIS-16 Versus PROMIS-29 Estimated Mental Health Summary Score

The Bland–Altman plot shows scatter bias for the difference between the PROMIS-16 and PROMIS-29 estimated physical health summary score, with the PROMIS-16 estimates overestimating the PROMIS-29 estimate at the upper (better health) end of the distribution (Fig. 1). Scatterbias is also seen for the PROMIS-29 mental health summary score (Fig. 2), but it is not as large as was seen for the physical health summary score.

### Correlates of mean differences between corresponding physical and mental health summary scores estimated by PROMIS-29 and PROMIS-16

Product-moment correlations indicated significant variation in differences between PROMIS-29 and PROMIS-16 estimated physical health summary scores by age (r = −0.03, p = 0.0398), female gender (r = −0.04, p = 0.0160), education (r = 0.07, p < 0.0001), and income (r = 0.11, p < 0.0001). Differences in PROMIS-29 and PROMIS-16 estimated mental health summary scores varied significantly by female gender (r = −0.04, p = 0.0098), education (r = 0.04, p = 0.0194), and income (r = 0.09, p < 0.0001). While different statistically at p < 0.05, these differences are small.

We illustrate the magnitude of the differences in the PROMIS-29 and PROMIS-16 estimates with income, the sociodemographic variable with the strongest association with the differences. Table 4 provides mean summary score differences by four levels of income. The PROMIS-16 physical health means were larger than the PROMIS-29 means among those with lower income and smaller for those with the highest income. All these mean differences are small.

**Table 4:** Mean T-score Differences Between PROMIS-16 and PROMIS-29 Estimated Physical and Mental Health Summary Scores by Family Income

Summary Score	< $10,000 (n = 112)	$10,000–49,999 (n = 964)	$50,000–99,000 (n = 1223)	> = $100,000 (n = 1705)	Overall (n = 4004)
Physical health	0.87	0.42	0.07	−0.20	0.06
Physical health with pain intensity	0.77	0.35	0.03	−0.22	0.02
Mental health	0.06	−0.003	−0.18	−1.36	−0.21
Mental health with pain intensity	−0.10	−0.10	−0.25	−0.40	−0.27

## Discussion

This study supplements prior work showing that the eight profile scores estimated from the PROMIS-16 have strong psychometric properties and are comparable to those estimated from the PROMIS-29 + 2 [6]. The results of these analyses of data from a large probability-based internet sample provide strong support for the equivalence of PROMIS-16 and PROMIS-29 estimates of the PROMIS physical and mental health summary scores. Moreover, findings for the PROMIS-16 summary scores are comparable across versions with and without the pain intensity item.

The PROMIS-16 reduces the response burden for research participants and patients relative to the PROMIS-29. Although there is a tradeoff in the precision of measurement for the two-item PROMIS-16 scales versus the four-item PROMIS-29 scales, the reliability of the physical and mental health summary scores estimated from the PROMIS-16 exceeds the 0.90 reliability threshold [13] for the use of measures for individual assessment. Hence, clinicians can obtain a precise assessment to monitor individual patients’ physical and mental health.

The use of a probability-based panel representative of the U.S. population is a strength of the study. However, some caution is needed in generalizing the results. Internet panel members tend to be more educated and have higher socioeconomic status than the general population [19]. Moreover, internet panelists may respond differently than others because of their ongoing opportunities to complete surveys. Finally, the results are limited to those who could complete the English-language survey.

Consistent with previous research [14] the PROMIS-16 and PROMIS-29 mental health summary scores correlated more strongly with the PROMIS global physical health score than with the PROMIS global mental health score. The high correlation occurs because of content overlap between the PROMIS-29 mental health summary score and the PROMIS global physical health scale: the PROMIS-29 fatigue scale has the largest and the pain composite the fourth largest factor scoring coefficient on the PROMIS-29 mental health summary score and two of the four PROMIS global physical health items are ratings of pain and fatigue.

Research is needed to evaluate the reliability and validity of the PROMIS-16 compared to the PROMIS-29 in different samples and among those with a variety of health conditions. Further examination of subgroup differences in agreement between PROMIS-16 and PROMIS-29 estimated physical and mental health scores could provide important information. Studies that randomize respondents to either the PROMIS-16 or PROMIS-29 would be useful to evaluate whether the response rate is higher for the more parsimonious measure. Head-to-head comparisons of the PROMIS-16 with the PROMIS global health scale would also be valuable to provide further guidance about the tradeoffs in using one versus the other.

## Data Availability

The data set analyzed for this study is publicly available from the ICPSR database repository number openicpsr-198049.
