# Correction: The PROMIS-16 reproduces the PROMIS-29 physical and mental health summary scores accurately in a probability-based internet panel

**DOI:** 10.1007/s11136-025-04093-9

**Published:** 2025-11-06

**Authors:** Ron D. Hays, Patricia M. Herman, Anthony Rodriguez, Mary Slaughter, Chengbo Zeng, Maria Orlando Edelen

**Affiliations:** 1 Division of General Internal Medicine and Health Services Research, UCLA Department of Medicine, 1100 Glendon Avenue, Suite 850, Los Angeles, CA 90024 USA; 2 RAND Corporation, Behavioral and Policy Sciences, 1776 Main Street, Santa Monica, CA USA; 3 RAND Corporation, Behavioral and Policy Sciences, 20 Park Plaza #910, Boston, MA USA; 4 Patient Reported Outcomes, Value and Experience (PROVE) Center, Department of Surgery, Brigham and Women’s Hospital, Boston, MA USA

**Correction to: Quality of Life Research (2025) 34:19–26** 10.1007/s11136-024-03662-8

In this article, the data in the first column of Table 2 were misaligned. The incorrect and corrected versions of Table 2 are provided in this correction.

Incorrect version of Table 2:

**Table 2:** Product-moment Correlations Among the PROMIS-16 and PROMIS-29 Measures

PROMIS-16								PROMIS-29							
PF	SO	EM	FT	SD	PN	PS	MS	PF	SO	EM	FT	SD	PN	PS	MS

PROMIS-16																
Rel	90	83	93	86	73	91	92	95	94	95	95	94	87	95	94	98
SO	57															
EM	29	59														
FT	44	65	60													
SD	32	54	52	57												
PN	66 (65)	62 (59)	40 (39)	52 (52)	45 (47)											
PS	99 (99)	67 (67)	36 (36)	51 (51)	38 (39)	74 (73)										
MS	54 (54)	85 (85)	80 (80)	89 (89)	71 (71)	69 (69)	64 (64)									

PROMIS-29																
PF	**95**	62	33	48	36	70 (68)	95 (95)	59 (59)								
SO	61	**94**	59	67	56	65 (62)	70 (70)	85 (84)	66							
EM	29	61	**97**	62	53	40 (40)	36 (36)	80 (80)	33	60						
FT	45	66	61	**96**	58	53 (54)	52 (52)	89 (89)	49	68	63					
SD	33	51	52	57	**81**	43 (45)	39 (39)	68 (68)	35	52	53	59				
PN	65	59	40	52	47	**92 (99)**	72 (73)	67 (69)	68	63	40	55	46			
PS	94	70	38	54	42	75 (74)	**96 (96)**	67 (67)	99	74	39	55	41	75		
MS	55	83	78	88	70	69 (70)	67 (64)	**98 (98)**	60	85	80	91	72	70	68	

*PF* Physical function, *SO* Ability to participate in social roles and activities, *EM* emotional distress, *FT* Fatigue, *SD* Sleep disturbance, *PN* Pain, *PS* Physical Health Summary Score, *MS* Mental Health Summary Score, *Rel*. Reliability estimates with only the two digits to the right of the decimal point shown.

Correlations are presented as if each measure was scored in the same direction and only the two digits to the right of the decimal point are shown. Bolded entries indicate correlations between corresponding PROMIS-16 and PROMIS-29 measures.

Correlations with PROMIS-16 including pain composite (average of pain interference and pain intensity) instead of just pain interference are provided within parentheses.

Correct version of Table 2:

**Table 2:** Product-moment Correlations Among the PROMIS-16 and PROMIS-29 Measures

	PROMIS-16								PROMIS-29							
	PF	SO	EM	FT	SD	PN	PS	MS	PF	SO	EM	FT	SD	PN	PS	MS
PROMIS-16																
Rel.	90	83	93	86	73	91	92	95	94	95	95	94	87	95	94	98
SO	57															
EM	29	59														
FT	44	65	60													
SD	32	54	52	57												
PN	66 (65)	62 (59)	40 (39)	52 (52)	45 (47)											
PS	99 (99)	67 (67)	36 (36)	51 (51)	38 (39)	74 (73)										
MS	54 (54)	85 (85)	80 (80)	89 (89)	71 (71)	69 (69)	64 (64)									
PROMIS-29																
PF	**95**	62	33	48	36	70 (68)	95 (95)	59 (59)								
SO	61	**94**	59	67	56	65 (62)	70 (70)	85 (84)	66							
EM	29	61	**97**	62	53	40 (40)	36 (36)	80 (80)	33	60						
FT	45	66	61	**96**	58	53 (54)	52 (52)	89 (89)	49	68	63					
SD	33	51	52	57	**81**	43 (45)	39 (39)	68 (68)	35	52	53	59				
PN	65	59	40	52	47	**92 (99)**	72 (73)	67 (69)	68	63	40	55	46			
PS	94	70	38	54	42	75 (74)	**96 (96)**	67 (67)	99	74	39	55	41	75		
MS	55	83	78	88	70	69 (70)	67 (64)	**98 (98)**	60	85	80	91	72	70	68	

*PF* Physical function, *SO* Ability to participate in social roles and activities, *EM* emotional distress, *FT* Fatigue, *SD* Sleep disturbance, *PN* Pain, *PS* Physical Health Summary Score, *MS* Mental Health Summary Score, *Rel*. Reliability estimates with only the two digits to the right of the decimal point shown.

Correlations are presented as if each measure was scored in the same direction and only the two digits to the right of the decimal point are shown. Bolded entries indicate correlations between corresponding PROMIS-16 and PROMIS-29 measures.

Correlations with PROMIS-16 including pain composite (average of pain interference and pain intensity) instead of just pain interference are provided within parentheses.

The original article has been corrected.

